# Acute Clinical Manifestation of Mesenteric Heterotopic Pancreatitis: A Pre- and Postoperative Confirmed Case

**DOI:** 10.1155/2018/5640379

**Published:** 2018-04-15

**Authors:** Bente M. de Kok, Fleur I. de Korte, Lars E. Perk, Valeska Terpstra, J. Sven D. Mieog, Frank M. Zijta

**Affiliations:** ^1^Department of Radiology, Haaglanden Medical Centre, Lijnbaan 32, 2512 VA The Hague, Netherlands; ^2^Department of Gastroenterology, Haaglanden Medical Centre, Lijnbaan 32, 2512 VA The Hague, Netherlands; ^3^Department of Pathology, Haaglanden Medical Centre, Lijnbaan 32, 2512 VA The Hague, Netherlands; ^4^Department of Surgery, Leiden University Medical Centre, Albinusdreef 2, 2333 ZA, Leiden, Netherlands

## Abstract

Heterotopic pancreas is a relatively uncommon congenital anomaly, defined as pancreatic tissue in ectopic sites without an anatomic and vascular continuity with the main body of the pancreas. We report the case of a 58-year-old woman who was admitted to the hospital with the clinical suspicion of a mild, acute pancreatitis. Computed tomography, magnetic resonance imaging, transabdominal ultrasound, and endoscopic ultrasound revealed a normal orthotopic pancreas and the suspicion of a large heterotopic pancreas in the small bowel mesentery with signs of acute inflammation. The diagnosis of mesenteric heterotopic pancreatitis was preoperatively confirmed by endoscopic ultrasound-guided fine-needle aspiration and consequently histologically established after surgical resection.

## 1. Introduction

Heterotopic pancreas, also referred to as ectopic pancreas, aberrant pancreas, or choristoma, is defined as the presence of pancreatic tissue in ectopic sites which lacks an anatomic and vascular continuity with the main body of the orthotopic pancreas. Although it is the most common heterotopia within the gastrointestinal system, it still concerns a relatively uncommon entity with prevalence at autopsy studies ranging from 0.6 to 13.7% [1]. Most prevalent anatomic sites include the stomach, duodenum, and jejunum, whereas a manifestation in the small bowel mesentery is fairly rare. Though the majority of patients are asymptomatic, with lesions being incidentally detected at unrelated surgery or autopsy, patients with heterotopic pancreas may present with clinically significant pathology, identical to the normal pancreas.

## 2. Case Report

A 58-year-old woman presented to the Emergency Room with an acute, 4-hour history of increasing continuous abdominal pain. The pain was severe and associated with nausea and vomiting. She reported not having a medical history, no history of a similar episode of abdominal pain, no alcohol consumption, and no smoking. Physical examination revealed generalized abdominal pain. The vital signs were stable. Early laboratory values included a normal white blood cell count (8.8*∗*10^9^/L) and a normal C-reactive protein (CRP) level (3 mg/L, reference range 0–8 mg/L). Serum amylase was increased (417 U/L; reference value < 107 U/L). Liver function tests results were within normal ranges. Besides a significantly increased urine amylase (2286 U/L, reference range 0–460 U/L), urinalysis was normal. Additionally, abdominal ultrasound was performed but the diagnostic value was limited because of body habitus. The ultrasound examination revealed a small amount of fluid around the liver. There was no evidence of cholelithiasis. The pancreas was not conclusively visualized. With the suspicion of a mild, acute pancreatitis, based on both clinical and laboratory findings, the patient was admitted to the hospital for conservative treatment and both biochemical and radiologic follow-up.

During admission, the abdominal pain persisted despite pain medication and the level of CRP increased, reaching its maximum of 580 mg/L on day 3. According to the hospital protocol, an abdominal computed tomography (CT) was performed on the third day after onset.

**Figure 1:**
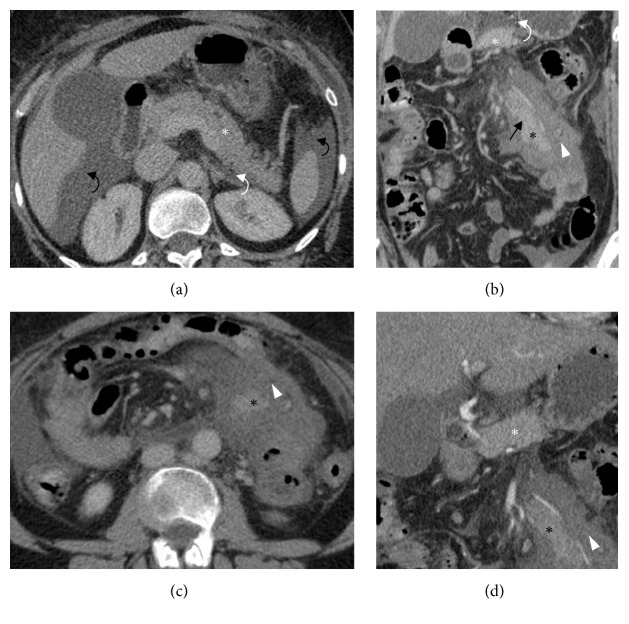
CT scan performed at the third day of admission. (a) Axial CT image in the portal venous phase. Normal appearance of the orthotopic pancreas* (white asterisks)*. A nonsignificant small amount of retroperitoneal fluid on the level of the pancreas tail* (white curved arrows)*. Presence of a large amount of free intraperitoneal fluid* (black curved arrows)*. (b-c) Coronal and axial CT image in the portal venous phase. Enhancing soft tissue mass* (black asterisk) *with surrounding mesenteric edema* (white arrowhead)*. Suggestion of a central ductal structure* (black arrow)*. (d) Coronal CT image in the early arterial phase shows a comparable enhancement of the orthotopic and heterotopic pancreas* (resp., white and black asterisks)*.

CT revealed a normal morphology and homogenous enhancement of the pancreas, with only a small amount of fluid located around the tail of the gland (Figure 1(a)). The presence of free intraperitoneal fluid was observed in the posterior right subhepatic space, left subphrenic space, paracolic spaces, and pelvis. An enhancing soft tissue mass in the centre of the jejunal mesentery was observed with adjacent increased attenuation of the mesenteric fat and enlarged surrounding lymph nodes. The mass measured up to 8 centimeters in long axis and demonstrated a lobulated morphology. Enhancement characteristics were comparable with normal pancreas enhancement. Also, the presence of a central ductal, tubular structure was suggested (Figures 1(b)–1(d)). Based on both imaging findings and laboratory results, the suspicion of a heterotopic pancreatitis was proposed.

**Figure 2:**
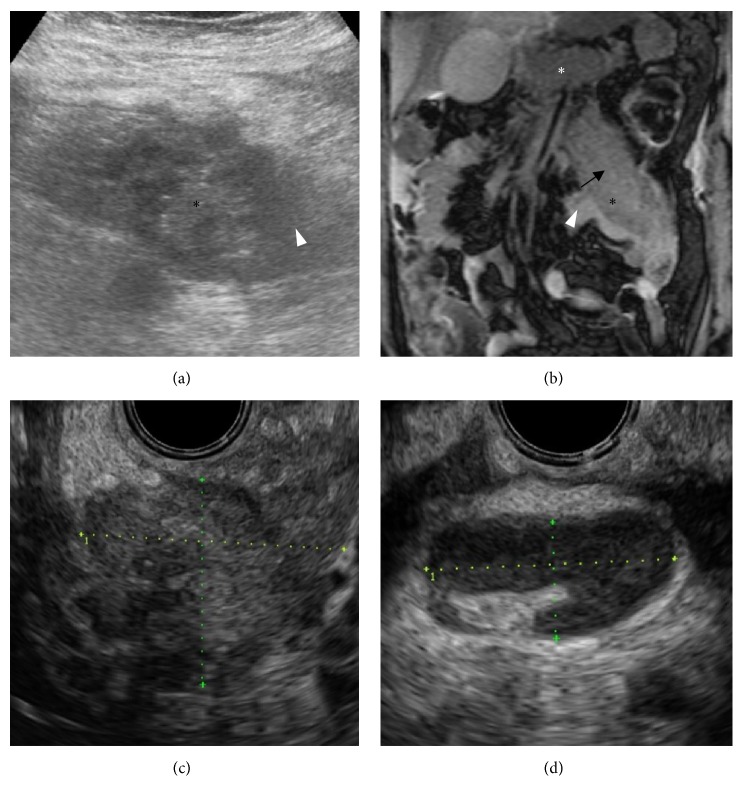
(a) Ultrasound image of the mesenteric soft tissue mass* (black asterisks) *with surrounding inflammatory changes* (white arrowhead)*. (b) Coronal TRUFI image depicting the soft tissue mass with signal characteristics according to the orthotopic pancreas* (white asterisk)*. Surrounding, edematous inflammatory changes* (white arrowhead)* and a possible central duct* (black arrow)*. (c) EUS image of the hypoechoic mesenteric soft tissue mass, in the transversal plane measuring approximately 3 × 4 cm. (d). EUS image of an enlarged mesenteric lymph node, with a short axis of 9 mm, demonstrating no suspicious morphological features.

To confirm the hypothesis of heterotopic pancreatitis, an additional targeted ultrasound and magnetic resonance imaging (MRI) of the abdomen were performed. Ultrasound depicted a nonspecific hypoechoic region in the mesentery with a central, lobulated, unsharp delineated, more hyperechoic soft tissue mass (Figure 2(a)). MRI with limited series was performed, including a fat-saturated gradient echo sequence TRUFI (true fast imaging with steady-state free precession) in the coronal plane, an ultrafast gradient echo (VIBE) in the axial plane, and an in-phase and out-of-phase gradient echo sequence in the axial plane. Quality of both axial sequences was limited due to motion-related artefacts in the ill, painful patient. No chemical shift artefacts supporting the heterotopic pancreas-hypothesis were apparent. The coronal TRUFI revealed a mesenteric soft tissue mass with signal characteristics resembling the orthotopic pancreas, surrounding inflammatory changes, and a possible duct (Figure 2(b)); however, these findings were noncontributory for the diagnosis. Additional laboratory findings included a normal carcinoembryonic antigen (CEA) and Cancer Antigen 19.9 level. The Cancer Antigen 125 (CA-125) level was 208 (normal range 0–35 kU/l). Yet both pancreatic cancer and pancreatitis can cause a high level of CA-125; therefore, this was regarded as an unspecific, nondifferentiating finding.

In order to rule out a malignancy and to confirm the diagnosis of heterotopic pancreatic tissue, the gastroenterologist performed an endoscopic ultrasound (EUS) with additional fine-needle aspiration (FNA). EUS performed from the horizontal part of duodenum showed a hypoechogenic mass of 3 × 4 cm with indistinct delineation and poor vascularisation with adjacent enlarged lymph nodes and inflammatory changes (Figures 2(c) and 2(d)). Endoscopic ultrasound-guided FNA of the soft tissue mass and the largest surrounding lymph node was performed. Cytopathologic examination revealed benign cells; the soft tissue mass was classified as pancreatitis not otherwise specified (NOS). There were no preexisting pancreatic cells distinguishable due to the inflammation; the lymph node was classified as lymphadenitis accordingly.

**Figure 3:**
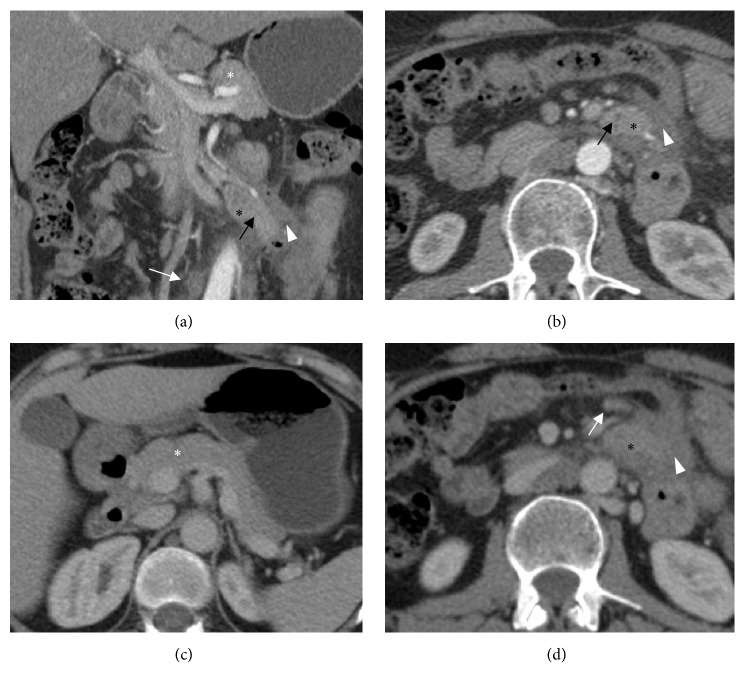
CT scan performed six weeks after admission. (a-b) Coronal and axial image in the arterial phase. (c-d) Axial images in the portal venous phase. Intra-abdominal free fluid is completely resolved. Heterotopic pancreas* (black asterisks)* in the mesentery with attenuation characteristics after intravenous contrast comparable to the orthotopic pancreas* (white asterisks)*. Moreover, presence of a central duct is suggested* (black arrow)*. There is an apparent decrease in inflammatory changes* (white arrowheads)* surrounding the heterotopic pancreas, though several adjacent mesenteric lymph nodes have increased in size* (white arrows)*.

After one week of conservative treatment, acute clinical findings resolved and laboratory results improved, consequently the patient was discharged from the hospital. A follow-up CT scan of the abdomen, six weeks after admission, revealed the soft tissue mass with substantial regression of inflammatory changes. There were no complications such as cyst formation or necrosis. The orthotopic pancreas was unremarkable (Figure 3).

**Figure 4:**
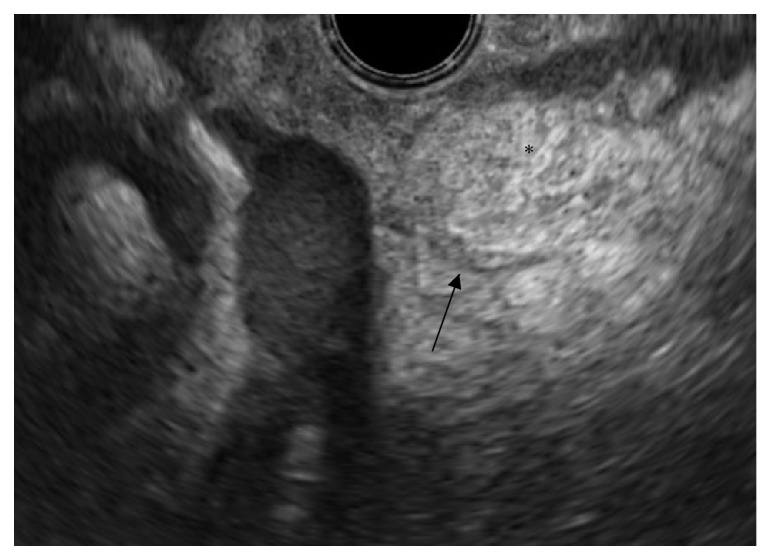
EUS image of the hyperechoic mesenteric soft tissue mass* (black asterisk)* with a hypoechoic, ductal structure* (black arrow)*.

**Figure 5:**
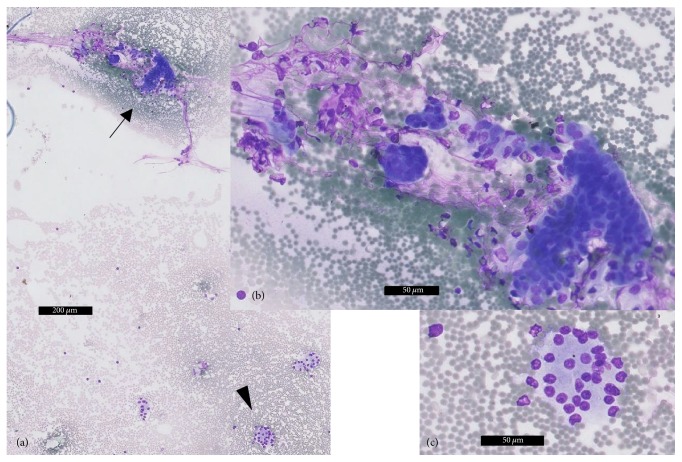
Fine-needle aspirate of the mesenteric soft tissue mass, MGG staining. (a) Overview, magnification 50x. Both groups of ductal cells* (black arrow)* and acinar cells* (black arrowhead)* are depicted in this image. (b) Magnification 200x, details of the cylindrical ductal cells. (c) Magnification 200x, details of the acinar pancreatic cells.

To conclusively validate the earlier clinical hypothesis and to further analyze the lymph nodes that had increased in size, an additional EUS was executed. EUS performed from the horizontal part of the duodenum depicted the pancreas-like mass with apparent ductal structures (Figure 4). The mass is evidently more hyperechoic than during the episode of inflammation (Figure 2(c)). No definite dominant duct was recognized, and there were no suspicious lymph nodes. Furthermore, FNA of the pancreas-like tissue was repeated. Cytopathologic examination confirmed the hypothesis of heterotopic pancreas showing pancreatic epithelium with both acini and ducts (Figure 5).

**Figure 6:**
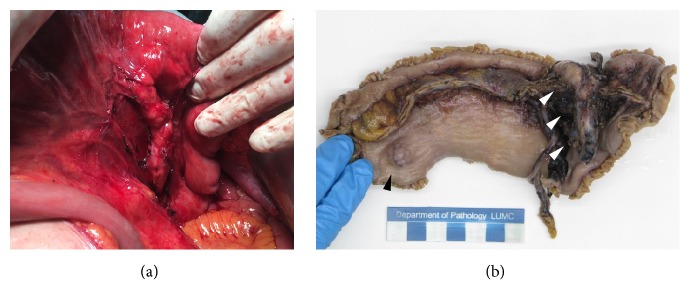
(a) Intraoperative view on the proximal jejunum and its mesentery showing the heterotopic pancreas extending centrally. (b) Gross specimen of the segment resection after the jejunum is opened, demonstrating the heterotopic pancreas* (white arrowheads)* connected to the jejunum and the second manifestation of heterotopic pancreas in the serosa of the jejunum* (black arrowhead)*.

The diagnostic findings and the possibility of a resection were discussed with the patient. Given the risk of recurrent episodes of pancreatitis and consequently more challenging surgery and possible malignant degeneration [1–4], the multidisciplinary team advised on a resection of the heterotopic pancreas. The patient followed this advice and underwent surgery. Through a midline incision the heterotopic pancreas was identified in the mesentery of the proximal jejunum approximately 5 cm distally of the ligament of Treitz (Figure 6(a)). The ligament was ligated, and the distal duodenum and proximal jejunum were mobilised. The heterotopic pancreas extended to the central mesenteric root, of which it could be dissected without compromising the mesenteric vascular supply. From a few centimeters of the jejunum, the mesenteric vessels were ligated, and a segment resection of the proximal jejunum was performed. The resection included a second spot of presumed heterotopic pancreas at the antimesenteric side of the jejunum, several centimeters distally of the primary heterotopic pancreas (Figure 6(b)). An enlarged mesenteric lymph node was sent for intraoperative frozen section analysis and was reported as benign. A primary end-to-end anastomosis was constructed. The postoperative course was uneventful. Pathological analysis revealed two locations of heterotopic pancreas of 6.5 cm and 1.5 cm with minimal signs of pancreatitis. The heterotopic pancreas contained acini, ducts, and islet cells.

## 3. Discussion

Generally considered a developmental anomaly, heterotopic pancreas is defined as anatomically separated pancreatic tissue from the main gland, without vascular or ductal continuity. Most cases of heterotopic pancreas are found in the upper gastrointestinal tract with 70–87% of cases affecting the stomach, duodenum, and jejunum in mostly submucosal localization [1, 5]. Less common sites of origin include Meckel diverticulum, ileum, gallbladder, bile ducts, Fallopian tubes, mediastinum, oesophagus, spleen, omentum, and mesentery [2, 5, 6]. Zhang et al. [7] and Henry et al. [6] report the mesenteric manifestation in, respectively, 3.8% and 5.9% of the heterotopic pancreas.

The exact embryologic basis of heterotopic pancreas is unknown, but there are three apprehended theories. The most widely held theory is the misplacement theory, which states that deposits of pancreatic tissue are “dropped” into the developing gastrointestinal system [2, 8, 9]. This would explain why the majority of cases of heterotopic pancreas are found in other derivatives of the primitive foregut. The two other theories include the metaplasia and the totipotent cell theory, which state the migration with transformation and, respectively, differentiation of endodermal cells into pancreatic tissue [2, 5, 9]. Histologically the heterotopic pancreas mimics the orthotopic pancreas. Gross specimens demonstrate a firm mass with a lobular shape and a well-defined interface with surrounding tissues. Microscopically, the heterotopic mesenteric pancreas infiltrates the bowel wall and extends into the submucosal layer. Four types of heterotopic pancreas are described in the histologic classification system by Fuentes (1973). The first and most common type of heterotopic pancreatic tissue is composed of all the elements of normal pancreas, including acini, ducts, and islet cells. The second, third, and fourth are dominated by, respectively, acini, ducts, and islet cells [2]. Based on histopathologic findings, our patient was classified as a type 1 heterotopic pancreas, containing acini, ducts, and islet cells.

It has been reported that the enhancement pattern of heterotopic pancreas varies depending on the microscopic composition and therefore can differ from the main pancreas. This can cause an unreliable differentiation from other homogenous enhancing tumours, such as gastrointestinal stromal tumour (GIST), carcinoid, lymphoma, or metastasis [10, 11]. Nevertheless, several characteristic CT features of heterotopic mesenteric pancreas resemble the orthotopic pancreas; these include a homogenous, well-enhancing lobulated mass with an elongated appearance. Most mesenteric manifestations have a close relation to the jejunum. The presence of a duct allows differentiation of manifest heterotopic pancreas from other masses [11]. The ductal system communicates with the bowel lumen, which might be difficult to visualize at various imaging modalities. MR cholangiopancreatography (MRCP) is the imaging method of choice to depict an ectopic duct, pathognomonic of ectopic pancreas [5, 12]. Unfortunately, in our patient no MRCP was performed. Possibly partly because of that, the MRI was not fully conclusive. Other contributing factors for the interpretability might have been motion artefacts and the performance in the acute phase, edema impeding identification of an apparent ductal morphology.

Most heterotopic pancreatic lesions exist solitary, in contrast with both the jejunal and mesenteric manifestation in our case, and measure smaller than 3.0 cm. A heterotopic mesenteric manifestation has a mean long axis diameter of 4.4 cm. It typically presents as an elongated lobulated lesion (long- to short-diameter ratio of 3.0) showing a broad base on the jejunal mesenteric side, tapering as it extends centrally into the mesentery [2, 11]. To our knowledge, the heterotopic pancreas in our case is with a long axis diameter of 8 cm on CT, the longest mesenteric pancreas described. In the acute phase, the heterotopic pancreas was swollen due to edema, and the gross specimen measured 6.5 cm. Shin et al. [8] described a patient with a 20 cm diameter heterotopic mass in the jejunal mesentery with a conglomerate of adjacent loops of jejunum, presenting with gastrointestinal bleeding. The article did not appoint the true size of the mass at histologic examination.

Characteristically, the majority of patients are asymptomatic, with lesions being incidentally detected at surgery, endoscopy, or autopsy. Complications of heterotopic pancreas may or may not be related to pathologic conditions of the pancreas itself. Those related might present with inflammation, (pseudo)cyst formation, endocrine (dys)function, or malignant degeneration [3, 4, 7, 9, 10, 13–17]. The pathologically unrelated cases might present with mechanical obstruction, intussusception, or bleeding [2, 8, 13].

Heterotopic pancreatitis is a recognized complication that is usually only detected at microscopy and is rarely diagnosed clinically or radiologically, especially in cases of submucosal manifestations. Usually, the elevation of serum amylase and lipase is modest due to the small volume of pancreatic tissue in the heterotopic tissue [5]. In our case, a relatively large heterotopic volume with clinically significant elevated serum amylase and C-reactive protein levels facilitated a preoperative clinical diagnosis. It must be noted though that the second serosal localization, which was depicted intraoperatively, was not depicted on preoperative imaging.

In case of an asymptomatic patient and histopathologically confirmed diagnosis, a conservative management might be applicable. However, in case of a symptomatic patient or if accurate differentiation from other benign or malignant aetiologies cannot be accurately made, curative resection of the heterotopic pancreas is usually considered [5]. The patient in this case was obviously symptomatic and had been admitted to the hospital for one week. In view of a potential recurrent pancreatitis and the low but not excluded risk of a malignant degeneration, both the patient and the multidisciplinary team decided on surgical resection [3, 4, 9].

## 4. Conclusion

Heterotopic pancreas is a relatively uncommon congenital anomaly. Most cases concerning solitary lesions were found in the upper gastrointestinal tract in submucosal localization. Though the majority of patients are asymptomatic, patients may present with clinically significant pathology such as inflammation or malignant degeneration, identical to the normal, orthotopic pancreas. We report a case of symptomatic, heterotopic pancreatitis in a patient with heterotopic pancreas tissue in the jejunal serosa and the jejunal mesentery. The clinical diagnosis was made using imaging modalities and cytopathological analysis, that is, ultrasound, CT, MRI, and EUS combined with FNA. Cytopathological examination preoperatively confirmed the diagnosis which is only limitedly reported in current literature. Subsequently it was histopathologically confirmed after complete resection.
